# Development of an Educational Video for Self-Assessment of Patients with RA: Steps, Challenges, and Responses

**DOI:** 10.31138/mjr.32.1.66

**Published:** 2021-03-04

**Authors:** Nelly Ziade, Thurayya Arayssi, Bassel Elzorkany, Amani Daher, Ghada Abi Karam, Mohammad Abu Jbara, Alla Aiko, Elie Alam, Samar Al Emadi, Manal Al Mashaleh, Humeira Badsha, Lina El Kibbi, Hussein Halabi, Ghita Harifi, Bhavna Khan, Abdel Fattah Masri, Jeanine Menassa, Mira Merashli, Georges Merheb, Jamil Messaykeh, Kamel Mroue’, Sahar Saad, Nelly Salloum, Imad Uthman, Basel Masri

**Affiliations:** 1 Saint-Joseph University, Beirut, Lebanon; 2 Weill Cornell Medicine, Doha, Qatar; 3 Cairo University, Cairo, Egypt; 4 Al-Bashir Hospital, Amman, Jordan; 5 Heartbeat Clinic, Beirut, Lebanon; 6 Levant Hospital, Beirut, Lebanon; 7 Hamad Medical Corporation, Doha, Qatar; 8 King Hussein Medical Center, Amman, Jordan; 9 Dr Humeira Badsha Medical Center, Dubai, United Arab Emirates; 10 Specialized Medical Center, Riyadh, Saudi Arabia; 11 King Faisal Specialist Hospital, Jeddah, Saudi Arabia; 12 Mediclinic City Hospital, Dubai, United Arab Emirates; 13 American University of Beirut, Beirut, Lebanon; 14 Lebanese University, Beirut; 15 Holy-Spirit University, Kaslik, Lebanon; 16 Monla Hospital, Tripoli, Lebanon; 17 Al-Zahraa Hospital, Beirut, Lebanon; 18 Assiut University, Egypt & King Hamad University Hospital, Bahrain; 19 Z-Clinic, Beirut, Lebanon; 20 Jordan Hospital, Amman, Jordan

**Keywords:** Rheumatoid arthritis, patient education, patient empowerment, self-assessment, educational video, DAS-28, composite measures, treat-to-target

## Abstract

**Objectives:**

The primary objective was to develop an educational video to teach patients with rheumatoid arthritis (RA) self-assessment of their disease activity. Secondary objectives were to validate the video, identify the challenges in producing it, and the responses to these challenges.

**Methods:**

Rheumatologists from 7 Middle Eastern Arab countries (MEAC) discussed unmet needs in the education of patients with RA. They reviewed pre-existing educational audiovisual material and drafted the script for a new video in Arabic. The video was produced in collaboration with a technical team, then validated by patients using a standardized interview. At each step of production, challenges were identified.

**Results:**

Twenty-three rheumatologists from MEAC identified unmet needs in patients’ education. A video was produced, explaining the concepts of treat-to-target and showing a patient performing self-assessment using DAS-28. Sixty-two patients were interviewed for validation and found the video to be useful and easy to understand, albeit not replacing the physician’s visit. Most common challenges encountered included acceptance of patient empowerment, agreement on DAS-28 as composite measure, production of a comprehensible written Arabic text, and addressing the population cultural mix.

**Conclusion:**

Despite challenges, the video was well accepted among patients and can be used for clinical and research purposes. It is particularly useful in pandemic periods where social distancing is recommended.

## INTRODUCTION

Rheumatoid Arthritis (RA) is the most frequent chronic rheumatic inflammatory disease, with an estimated prevalence of 0.24 to 1.25% in the adult population. Untreated, RA may lead to severe progressive joint destruction, functional disability, and premature mortality.^1–3^

Over the last decade, the concept of treat-to-target (T2T) revolutionized RA management and improved disease prognosis.^4,5^ First published in 2010, the main pillars of the T2T recommendations are the definition of a treatment target, the assessment of disease activity using composite measures, the modification of treatment if the target is not achieved within a specific timeframe, and the consideration of individual patient characteristics and shared decision-making.^6,7^

The use of composite disease measures are critical to the success of T2T,^8,9^ and their use in daily practice is recommended by major rheumatology associations such as the American College of Rheumatology (ACR) and the European League Against Rheumatism (EULAR).^10,11^ Such measures include the widely used Disease Activity Score (DAS-28), or the more recent scales, the Clinical Disease Activity Index (CDAI), and the Simplified Disease Activity Index (SDAI).^12,13^ All three correlate almost linearly with each other and with impairment in physical function.^14,15^

Although helpful, collecting these indices regularly in daily practice by the healthcare providers is time-consuming. Thus, involving the patient in the self-assessment of their joint count may expedite the evaluation process during their clinic visits. Moreover, empowering the patient, through education, improves the perception of disease activity in-between visits and enhances treatment adherence.^16^

With the help of new technologies, several self-assessment tools were recently developed and are available through interactive e-health platforms, web applications, and smartwatches. They allow the monitoring of patients between consultations and assist in making more informed treatment decisions. They are becoming a cornerstone of the emerging telemedicine movement, especially during times where social distancing is recommended, such as the COVID-19 pandemic.^17–22^

However, the optimal format for teaching self-assessment to patients is still debated. Using audiovisual material, such as videos, is one effective method to convey complicated medical concepts in a simple and accessible manner to a layperson.^23^ Video-assisted patient education has also shown efficacy in modifying patient behavior, especially when using a model patient enacting a behavior.^23^ The video format is time-efficient, round-the-clock accessible, and allows the learners to proceed at their own pace.^24^

The National Rheumatoid Arthritis Society (NRAS) and the Cochin hospital’s team developed each an educational video in English and French, respectively, to teach RA patients self-assessment of their disease activity using DAS-28.^25,26^ The videos are around 20 minutes long and feature a healthcare provider explaining the T2T concept, followed by a patient performing self-assessment of 28 joints. To our knowledge, no such material was developed and adapted in the Arabic language.

The objective of our study is to develop an educational video, in Arabic, to teach patients with RA self-assessment of their disease activity using DAS-28. Secondary objectives are to validate the video, identify challenges in producing it, and to discuss responses to these challenges.

## MATERIALS AND METHODS

### Identification of unmet needs in the education of patients with RA

Rheumatologists (“study team”) from seven Middle Eastern Arab countries (MEAC: Bahrain, Egypt, Jordan, Lebanon, Qatar, Saudi Arabia, and the United Arab Emirates) were invited through emails to participate in the project. The study team met in-person during a regional rheumatology meeting to identify the unmet needs in patient education in the Middle East and to delineate the ideal formats to address these needs. The development of a culturally appropriate educational video was identified as a priority, and a roadmap was created for its production.

### Development of the patient education video

A list of evidence-based guiding principles (**Table 1**) was used to develop the video.^16,24,27–35^ Pre-existing educational audiovisual material was identified through a search of the literature, of patients’ associations’ web-sites and social media platforms. Two videos addressing self-assessment using DAS-28 were found and reviewed by the study team. The first is published online in English by the NRAS, and the second in French by the Cochin hospital’s team, under the auspices of the French Society of Rheumatology.^25,26^ The strengths and weaknesses of these videos, as well as the appropriateness for the Middle Eastern cultural setting, were discussed by the study team, a rheumatology nurse, and a medical student. Based on the discussion, a script for the video was drafted. The physical exam section was transcribed from the French language video, translated into Arabic by the study team, and then reviewed by a professional translator for clarity of the language. A patient with RA was then invited to participate in to be the model/ actress in the video. Then, with the help of a professional technical team (comprising a movie director and an editor, with a vast experience of filming in several Arab countries, and a qualified voice-over expert), the video scenario was planned. This included choosing the video setting, the movie cuts sequence, and the patient’s clothing. On the day of filming, a rheumatologist (NZ) and a nurse (NS) were present on site. The video was edited in a professional studio in the presence of a rheumatologist (NZ) and a medical student (AD).

**Table 1.** List of guiding principles for developing an educational video.

**Guiding Principle**	**Reference**
Patient education should be provided for people with inflammatory arthritis as an integral part of standard care in order to increase patient involvement in disease management and health promotion	EULAR^16^
Patient education in inflammatory arthritis should be delivered by competent health professionals and/or by trained patients, if appropriate, in a multidisciplinary team	EULAR^16^
Patients should be involved in the development, delivery and the assessment of the intervention	29,30
Patients should hear from other patients	30–32
Information aimed to promote intrinsic motivation (teaching, goal setting, etc.), will be incorporated as possible, as it may improve adherence	33,34
Education should be understandable for patients with low health literacy	35
Education should be culturally competent	27,31

### Evaluating the patients’ perceptions about the video

**Figure 1.**
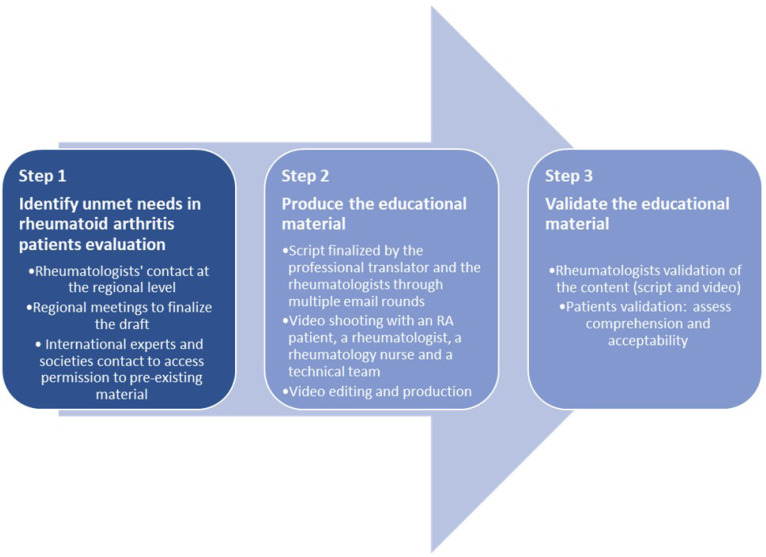
Flowchart of the successive steps of the educational video production: from designing to validation.

First, the video was reviewed for accuracy and clarity by the study team through multiple email rounds. Then, a sample of consecutive Arabic speaking patients with RA from 6 MEAC was invited by their rheumatologist to participate in evaluating the video. The rheumatologist explained the purpose of the video and obtained consent from the patients. In a separate room, the patients viewed the video and were interviewed by a trained rheumatology nurse who collected the patients’ feedback about the clarity of the video, acceptance, ability, and willingness to self-assess (**Figure 1**).

The study was approved by the Central Ethics Committee of Saint-Joseph University, Beirut, Lebanon, and by all local the Ethics Committees of the participating sites. All patients signed an informed consent form prior to enrollment in the study.

## RESULTS

### Identification of unmet needs in the education of patients with RA

Twenty-three rheumatologists from 7 MEAC participated in the study. All agreed that several gaps exist in patient education for rheumatic diseases, particularly for RA. The top two gaps identified were: 1) the patient’s difficulty in understanding the chronic nature of rheumatic diseases, and 2) the low adherence to prescribed regimens, especially after the symptoms have improved. Those gaps were deemed as a priority as they are tightly related to disease prognosis. Moreover, the study team agreed that, although the use of objective composite measures for assessment of disease activity in RA was desirable and can improve patients’ outcomes, their implementation can be highly time-consuming in daily practice. Therefore, developing appropriate educational material that involves patients in their disease management strategy was considered a priority by the study team.

The study team then discussed and resolved several challenges before proceeding into the stage of video development. The first was to decide on the choice of the disease activity measure to be used in the video. The following measures were discussed: DAS-28, CDAI, and SDAI. Although DAS-28 has some limitations, it was strongly recommended by the majority of the study team as it is the most widely used and includes joint counts, inflammatory markers, and patient’s global assessment.^36,37^

The second challenge was accepting the idea of patient empowerment and participation in their disease management. Several rheumatologists, especially those working in the private healthcare sector, raised their concern about whether promoting patient self-assessment could lead to a decrease in the frequency of patients visits and a decline in their income. This could be a valid concern, yet the purpose of promoting self -assessment is to complement the physician’s evaluation, decrease the visit time, and motivate the patient’s adherence to the therapeutic strategy.

The third challenge was the skepticism of some members of the study team about patients’ adherence to the skills taught in the video. It was agreed that the rheumatologist should use their judgment in selecting the suitable candidate-patient to whom self-assessment would be offered.

### Development of the patient education video

The two videos from NRAS and Cochin had detailed content and were used as reference documents. Both NRAS and Cochin team provided permission to use the videos for information. However, the study team felt that both videos were too long (around 20 minutes), that some sections used technical terms that may be difficult to understand, and that the choice of clothing of the model may not be acceptable by all cultures in the region. Also, both videos were relatively difficult to access, requiring an internet connection or a DVD player. Thus, the study team decided to create a new video in Arabic that would be shorter in duration, uses less technical terms, and better aligns with the local culture. Moreover, the video was designed in a user-friendly format and accessible without the need for an Internet connection.

The study team wrote the script for the video. The script was then reviewed by a professional translator for accuracy. To simplify the medical terms used in the video and make them understandable by the majority of the patients who speak the different Arabic dialects, the study team, using euphonic nomenclatures, translated these terms based on their dialect. Then, they reached a consensus on which translation should be included in the video.

The choice of the actor’s gender and clothing was also a matter of debate by the study team. Taking into consideration that the target audience is middle-aged women with diverse cultural and religious beliefs, a middle-aged female RA patient, with previous experience in television advertising, was selected for the role. The patient/actor wore neutral, conservative, and dark clothing for the filming. Prior to filming, she received a detailed briefing by her rheumatologist on self-assessment of disease activity. The video was filmed in a traditional home setting in the presence of a professional technical team, a rheumatologist, and a nurse. Then, the best video cuts were selected by the technical team. The cuts went through editing, voice cover, musical cover, and subtitling phases. The duration of the video was seven minutes, which was significantly shorter than the reference videos. English subtitles were prepared by the translator and reviewed and approved by the study team through multiple email rounds.

The new video was made easily transferable to the patient’s cellular phone and accessible without the need for an internet connection. The video can be accessed at this link: https://www.youtube.com/results?search_query=autodas+meac.

After completion of the video and in collaboration with a graphic designer, a supplementary cartoon version, depicting a male character, was also created as an alternative to be used for male patients and patients from more conservative populations.

### Evaluating the patients’ perceptions about of the video

After the study team approved the video, a sample of 62 consecutive RA patients attending the rheumatology clinics of the study team participated in the validation of the video. All patients were keen to be educated about their disease management and to understand the treatment decision algorithm followed by their physicians. However, several were reluctant to accept that they can accurately self-assess, and most stated that it would not replace the doctor’s visit. Ninety-six percent found the section of the video explaining the self-joint examination was easy to understand. Forty-one percent of the patients, however, found the scoring to be challenging and needed practice and support from the physician or the nurse. Most of the patients (76%) reported that self-assessment would not totally replace the doctor’s visit. Several challenges were identified during the development of the video and its validation (**Table 2**). First, it was challenging for patients to feel confident in the accuracy of their assessment. They were reassured that their self-assessment will not replace the rheumatologist’s assessment but rather than help them make more informed therapeutic decisions.

**Table 2.** Challenges and responses through the development of the video, at the different levels: rheumatologist, patient, cultural and logistics.

**Challenges**	**Responses**
***Rheumatologist’s level***	
Approve DAS-28 as a proper treatment target	Use DAS-28 as it was accepted by most rheumatologists, consider CDAI and SDAI for future steps
Accept the idea of patient empowerment	Use clinical judgment to choose the suitable patient to whom this education will be beneficial. Make a short, accessible, user-friendly video
Be skeptical about patient adherence to the use of the video	
***Patient’s leve**l*	
Accept the idea of patient empowerment	Conduct a qualitative validation with a sample of RA patients. Use clinical judgment to choose the suitable patient to whom this education will be offered and beneficial.
Understand some of the video parts on how to properly examine some joints	
Have a low confidence level about the scoring part	
***Cultural level***	
Produce a uniformly acceptable and comprehensible	Involve professional translators, rheumatologists, a medical student and a nurse in the script production, as well as patients for validation
Produce an educational video that is culturally acceptable written Arabic text despite different dialects	Obtain feedback from rheumatologists from different countries and different work settings Use adequate clothing for the video Produce an additional cartoon version
Address the population cultural mix in some countries across the different countries	
***Logistic level***	
Obtain non-biased funding source	Improvise study meetings in parallel to existing local, regional and international meetings Rely on the email system and WhatsApp group discussion

Second, several patients found the scoring to be challenging. This was facilitated by providing several practice sessions by the physician or study nurse that helped improve their skills and confidence.

Third, it was challenging to find an unbiased source of funding. Thus, to complete the project, all centers graciously volunteered to participate in the study without financial compensation. All study group meetings were aligned with local, regional, or international conferences.

## DISCUSSION

The production of an educational video in Arabic for self-assessment of disease activity for patients with RA was successfully implemented.

This was primarily facilitated by the collaboration between rheumatologists from several Arab countries, as witnessed in previous RA projects.^38^ Additional important enablers were adhering to the evidence-based guiding principles for developing educational videos^16,24,28^ and identifying the unmet needs for RA patient education by the study group.^39,40^ Moreover, the availability of two previous videos^25,26^ allowed recognizing a priori strengths and weaknesses in each video, which helped improve the current video. Finally, the inclusion of patients in the validation phase added to its pertinence and applicability. A major advantage of the produced video was the collaboration between a highly experienced team of rheumatologists and a professional technical team. This collaboration lead to a script that was understandable in most Arab countries and visuals that are culturally appropriate to the Middle East region. The English subtitles made the video also accessible to non-Arab speaking expatriate patients living in Arab countries. Moreover, having a real patient acting in the video facilitated the acceptance by the target patients’ population.^23,24,28^

Furthermore, as compared to the Cochin and NRAS video, the video was relatively short in duration, downloadable on a smartphone, and accessible without the need of an internet connection, making it easier to watch repeatedly. Finally, if needed at a later stage, the video can be easily modified and adapted for use by other populations. The outcome of educational videos on several different topics, such as education about pneumococcal vaccination^41^ and cervical cancer health literacy,42 was a clear impact on changing patients’ behaviors positively. Also, the above videos, as well as the NRAS and Cochin RA videos,^26,43,44^ were well received by the patients who appreciated the education offered to them.

The video format is relatively expensive to produce, and its creation is time-consuming. However, it remains an accessible source of education to most of the patients and allows them to watch it repetitively at their own pace, from the comfort of their home.^24^

The study also has several limitations. The choice of DAS-28 as the outcome measure may be questioned.^36,45^ DAS-28 is strongly associated with both inflammation and patient-reported outcomes.^36^ However, the subjectivity related to the patient global assessment and the possible variability of the biological inflammatory markers, which may be elevated due to causes other than RA, may have an impact on the stability of the final score. Moreover, DAS-28 calculation may be complicated for the layperson and needs a special calculator. In case patients were resistant to calculating a composite score, the concordance of more intuitive scores, such as CDAI and SDAI, may be used as alternatives in the future. The video may be easily adapted to editing the final section on scoring.

Another limitation is the generalisability of the video to different patient populations. Patients with low levels of education may find the video difficult to use and, therefore, may not fully benefit from this educational intervention. Finally, patients who may be known to be non-compliant to treatment may use the self-assessment as an excuse to skip important and irreplaceable visits to the rheumatologist. Although this was not reflected in the patient interview, the clinical judgment of the rheumatologist should guide the selection of patients to whom the video will be offered and how it will be used. Empowering patients to self assess their disease activity might be viewed as a double-edged sword to some physicians. They might fear some loss of income by losing the visits of patients who can self-assess their disease. In our opinion, and in the setting of a busy practice, the precious time saved can provide additional consultation time slots to offer care for more patients in more need to see the rheumatologist, potentially increasing both income and patient satisfaction.

## CONCLUSION

The collaboration between several countries sharing the same language, close cultural backgrounds, and comparable unmet needs was possible and able to produce an educational material in lay language aiming at self-assessment of patients with RA. Many challenges were faced at every step of the production and were resolved through consensus and teamwork. The video may serve for future research and clinical purposes, according to the rheumatologist’s clinical judgment.
